# Fused Granular Fabrication of Biodegradable Lightweight Knee Orthoses: Printability of Poly(lactic Acid)/Poly(butylene Adipate-Co-Terephthalate)/Bamboo Fiber Composites

**DOI:** 10.3390/ma19194221

**Published:** 2026-10-05

**Authors:** Zixin Hu, Weiping Dong, Bin Wang, Zhenxiao Xu, Xiping Li, Sisi Wang, Peng Zhao

**Affiliations:** 1 College of Engineering, Zhejiang Normal University, Jinhua 321004, China; 2 Zhejiang Key Laboratory of Additive Manufacturing Technology and Equipment, School of Mechanical Engineering, Zhejiang University, Hangzhou 310058, China

**Keywords:** 3D printing, fused granular fabrication, poly(lactic acid), composites, orthoses

## Abstract

**Highlights:**

• Lightweight PLA/PBAT/BF orthoses successfully fabricated via FGF 3D printing;
• Optimal printing: 10 wt.% BF, 190–220 °C, 30 mm/s;
• Bamboo fiber enables tough, biodegradable, custom-fit orthoses.

**Abstract:**

Conventional knee orthoses (KOs) are fabricated from petroleum-based plastics and are difficult to recycle, while filament-based fused deposition modeling is limited by low deposition efficiency and weak interlayer strength for large load-bearing devices. Herein, the authors reported fused granular fabrication (FGF) of lightweight, biodegradable KOs from poly(lactic acid)/poly(butylene adipate-co-terephthalate)/bamboo fiber (PLA/PBAT/BF) composites. Composites with 2, 6, and 10 wt.% BF were melt-compounded using zinc acetate as a transesterification catalyst and directly granule-printed. Rheological and morphological analyses revealed that increasing BF loading raised melt viscosity and elasticity, albeit with local fiber aggregation. Tensile strength decreased from 37.0 to 26.0 MPa with fiber addition, whereas notched impact strength improved from 7.2 to 11.6 kJ/m^2^, attributed to fiber pull-out, interfacial debonding, and matrix plastic deformation. A demonstration KO weighing only 243 g was printed. Printability depended strongly on nozzle temperature and speed: at 40 mm/s, all BF loadings failed, whereas 10 wt.% BF achieved defect-free printing at 220 °C and 30 mm/s. An optimal processing window of 10 wt.% BF, 190–220 °C, and 30 mm/s yielded tough, lightweight, biodegradable, custom-fit orthoses, demonstrating promising potential for rehabilitation applications.

## 1. Introduction

Knee orthoses (KOs) are widely prescribed to patients with lower limb motor dysfunction caused by sports injuries, such as anterior cruciate ligament rupture and patellar dislocation [1,2]. These orthoses stabilize joints, correct deformities, and assist gait rehabilitation, thereby playing a critical role in preventing secondary injuries and facilitating return to sport. However, conventional KOs are predominantly manufactured from petroleum-based plastics (e.g., polypropylene, polyethylene) using thermoforming or hand-laminating techniques [3]. After their service life, these devices are difficult to recycle due to material contamination and mixed components, leading to accumulation in landfills or incineration that causes white pollution [4,5]. Therefore, developing a bio-based, degradable alternative for KO production is both environmentally urgent and clinically relevant.

Polylactic acid (PLA) is a biodegradable polyester derived from renewable resources (e.g., corn starch, sugarcane) [6,7]. Its high modulus and good printability make it attractive for orthotic applications, but its inherent brittleness limits its impact resistance [8]. Blending PLA with poly(butylene adipate-co-terephthalate) (PBAT), a flexible and tough biodegradable polymer, effectively toughens the PLA matrix by introducing a compatible interface [9,10]. Further reinforcement can be achieved by incorporating natural fibers [11]. Bamboo fiber (BF) is particularly promising because it is renewable, completely biodegradable, lightweight, and possesses high specific strength [12,13]. Moreover, the low density of BF enables significant weight reduction in the final orthosis, improving patient comfort and compliance during long-term wear.

Fused deposition modeling (FDM) using filament-based feedstock has been extensively explored for customizing KOs because of its low equipment cost and ease of operation [14]. Patient-specific digital models can be directly 3D printed, eliminating expensive molds. However, filament-based FDM suffers from two major limitations when fabricating large orthoses: low deposition efficiency due to the small nozzle diameter (usually 0.4 mm) and limited filament diameter tolerance [15]. Another major drawback is insufficient interlayer strength, especially under impact loading, because the layer-by-layer bonding relies primarily on thermal diffusion without applied pressure. These drawbacks hinder the widespread adoption of FDM for load-bearing orthotic devices that require both production speed and mechanical robustness.

To overcome these challenges, granule-based screw-extrusion 3D printing, known as fused granular fabrication (FGF), has emerged as an alternative additive manufacturing technology [16,17,18,19]. FGF directly uses commercial polymer pellets, eliminating filament-drawing steps and reducing material cost. The larger nozzle diameter and continuous screw-driven extrusion enable high deposition rates, considerably shortening printing time for KOs. Furthermore, the screw-driven extrusion process can provide continuous material deposition and may promote intimate contact between adjacent deposited layers through the pressure generated during extrusion [20].

Our previous work demonstrated the feasibility of using PLA/PBAT/BF composites for 3D-printed orthopedic braces and identified an optimized PLA/PBAT ratio and KH570 treatment condition [21]. However, the previous study mainly focused on material preparation and preliminary evaluation of composite processability. The present study investigates a different material design based on a PLA/PBAT (80/20) matrix containing zinc acetate and systematically examines the effects of BF loading on the mechanical properties of the composites. In addition, the effects of FGF processing conditions on the printability of customized KOs are evaluated through printing-parameter optimization and infrared thermography. The results demonstrate that FGF-printed KOs with optimized BF loading and processing parameters achieve lightweight performance based on a biodegradable polymer composite system, offering a sustainable and high-performance solution for customized orthotic applications.

## 2. Materials and Methods

### 2.1. Materials

The PLA (grade: Revode190) was purchased from Zhejiang HISUN Biomaterials Co., Ltd. (Taizhou, China). It has a number-average molecular weight (*M*n) of 140,000 g/mol and a melt flow rate (MFR) of 6.5 g/10 min (210 °C, 2.16 kg). The PBAT (A400, Zhuhai KINGFA Biomaterials Co., Ltd., Zhuhai, China) has an Mn of 40,000 g/mol and an MFR of 8.0 g/10 min (190 °C/2.16 kg). Raw BF was purchased from Jiangxi Jindu Fine Handicraft Art Co., Ltd. (Jian, China). The BF has an average diameter of 180 μm and an average aspect ratio of 15. Both zinc acetate and a silane coupling agent (KH570) were purchased from Shanghai Yuanfan Scientific Co., Ltd. (Shanghai, China).

### 2.2. Preparation of PLA/PBAT/BF Composites

**Figure 1:**
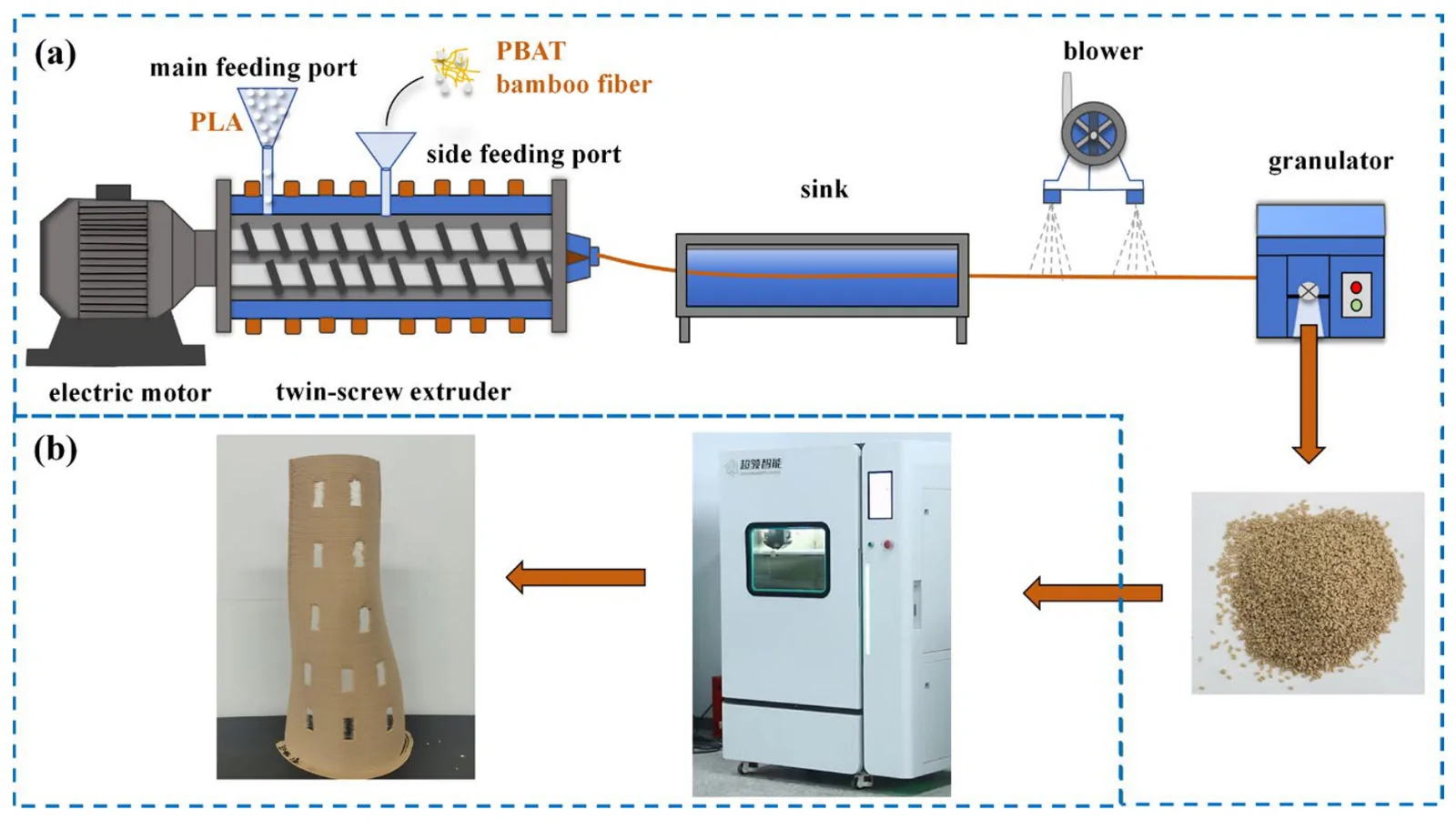
(**a**) Schematics of extrusion compounding of PLA/PBAT/BF composites, (**b**) FGF 3D printing of knee orthosis.

Before melt mixing, the BF was treated with the KH570 in a water bath at 60 °C for 2 h, and then was dried in a vacuum oven at 80 °C for 12 h. The melt mixing was carried out using a twin-screw extruder (SHJ-35, Nanjing Shengchi Rubber and Plastics Machinery Manufacturing Co., Ltd., Nanjing, China) with a diameter of 35 mm. As shown in Figure 1a, during melt mixing, the PLA and zinc acetate were fed into the extruder from the hopper, while the PBAT and KH570-treated BF were fed into the extruder using a side feeder. The temperature was set to 130, 170, 190, 190, 200, 190, 195, 195, 195 °C from hopper to die, and the screw speed was 280 r/min. The weight ratio of PLA and PBAT was fixed at 80/20, while three BF loadings were set at 2.0 wt.%, 6.0 wt.%, and 10 wt.%. The loading of zinc acetate was fixed at 0.03 wt.%. During melt mixing, the zinc acetate was expected to scissor the PLA molecular chain to obtain many shorter hydroxyl-terminated PLA chains, which would undergo transesterification with PBAT [22]. Thus, the PLA and PBAT could be compatibilized.

The as-extruded PLA/PBAT/BF composites were named as PLA/PBAT/BF-x, where x designates the BF loading. The image of PLA/PBAT/BF-6 pellets is selectively shown in Figure 1a. The obtained composites were thoroughly dried at 80 °C to remove residual moisture before subsequent processing. After that, the composites were compression molded into disk-shaped samples with a diameter of 25 mm and a thickness of 2 mm at 210 °C. The samples were used for rheological and SEM analysis.

### 2.3. FGF 3D Printing

The digital model of the KO was obtained by three-dimensional surface scanning of the first author’s knee-lower leg region and was then imported into CAD software (AutoCAD 2021) and converted to STL format. Slicing was then performed with a layer thickness of 1 mm and 100% infill density to generate G-code containing the nozzle travel paths. The as-extruded PLA/PBAT/BF composites were 3D printed into KOs using an FGF 3D printer (CI Uni-Print 4-axis, Zhejiang Challenge & Intelligent Co., Ltd., Jinhua, China), as is shown in Figure 1b.

The FGF 3D printer was equipped with a single-screw extrusion system, which could melt and plasticize the polymers to supply melts for deposition [16]. The diameter of the screw is 14 mm, while the diameter of the nozzle is 3 mm. To evaluate the 3D printing adaptability of PLA/PBAT/BF composites, three nozzle temperatures (170, 190, and 220 °C) and three different print speeds (20, 30, and 40 mm/s) were applied to print the KOs. Moreover, the as-extruded PLA/PBAT/BF composites were also 3D printed into standard tensile and impact samples for mechanical tests. The nozzle temperature was set at 190 °C, and the print speed was 50 mm/s. Specifically, dog-bone-shaped tensile specimens and rectangular impact specimens were 3D printed in a flat build orientation with a 0° raster angle. A V-notch with a depth of 2 mm was machined into the impact specimens using a notching machine.

### 2.4. Characterization

Dynamic oscillatory shear rheological measurements were conducted on an MCR302 rotational rheometer (Anton Paar Instrument, Shanghai, China). All tests were performed at 190 °C with a fixed strain amplitude of 0.8%, while the angular frequency was swept from 0.1 to 100 rad/s. The disk-shaped PLA/PBAT/BF composite samples were then loaded between parallel plates of 25 mm diameter with a gap set to 1 mm.

Scanning electron microscopy (SEM) was employed to investigate the fractured surfaces of PLA/PBAT/BF composites. Specimens cut from the disk-shaped samples were cryogenically fractured in liquid nitrogen and subsequently coated with gold via sputter deposition. Observations were then performed on a Sigma300 SEM instrument (Carl Zeiss, Oberkochen, Germany) at an accelerating voltage of 10 kV. The fractured surfaces of impacted samples were also examined via SEM after gold sputtering.

Tensile tests were conducted on a universal testing machine (UTM 4024, SUNS, Shenzhen, China) at a crosshead speed of 5 mm/min, according to ASTM D638-10, Type I. Izod-type cantilever-beam impact tests were performed on an impact tester (PTM7151, SUNS, Shenzhen, China) fitted with a 5.5 J pendulum hammer, according to ASTM D256. Both tensile and impact samples were fabricated by FGF 3D printing. To minimize measurement variability, a minimum of five specimens were tested per condition.

An infrared thermal imager (E09-Pro, HIKMICRO, Hangzhou, China) was used to monitor the surface temperature of molten PLA/PBAT/BF composites during deposition, with thermal images captured immediately after each layer was deposited. An emissivity of 0.95 was adopted for the composites, and the reflected apparent temperature was set to 25 °C. The camera was factory-calibrated without additional blackbody calibration and positioned 30 cm from the sample at 15° from above. The region of interest was defined on the first layer of the porous region, 3 cm from the left edge, to avoid edge heat accumulation. Five images were recorded at five different layers for each sample under identical conditions.

## 3. Results and Discussion

### 3.1. Linear Rheological Properties of PLA/PBAT/BF Composites

**Figure 2:**
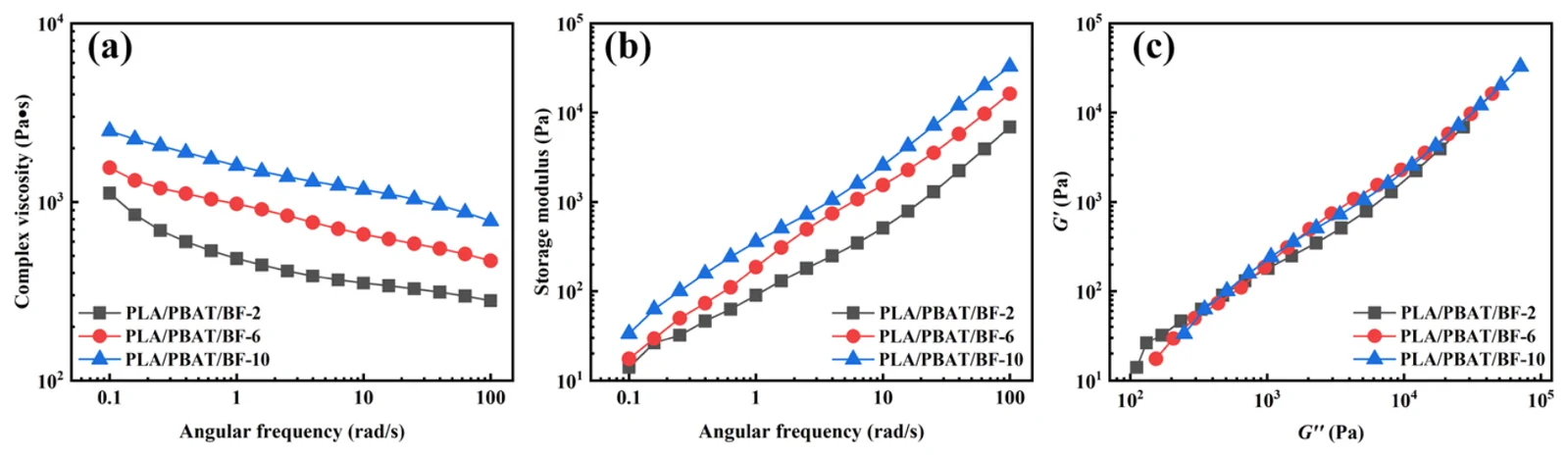
(**a**) Complex viscosity, (**b**) storage modulus, and (**c**) Han plots of PLA/PBAT/BF composites.

Figure 2 presents dynamic rheological measurements of PLA/PBAT/BF composites with different BF loadings. As shown in Figure 2a, all three samples exhibit typical shear-thinning characteristics, where complex viscosity declines with rising angular frequency. At a fixed frequency, complex viscosity increases sequentially from BF-2 to BF-10, because rigid BF segments restrict the mobility of polymer molecular chains and raise internal frictional resistance [23]. The storage modulus (G’) increases monotonically with angular frequency (Figure 2b), and higher BF loading produces elevated G’ across the whole frequency window, indicating enhanced melt elasticity after fiber incorporation.

The Han plots (lg G’ versus lg G”) can provide the information on filler dispersion. Although all curves follow similar overall trends, gradual upward deviation occurs with increased BF loading. This subtle divergence reflects the non-uniform distribution of BF within the PLA/PBAT matrix. The fibers form local concentrated domains rather than a fully homogeneous dispersion. This rheological feature is consistent with the expectation of filler aggregation at higher loading, while the similar slopes of the curves prove that the good miscibility of the PLA/PBAT blend matrix is not destroyed.

### 3.2. Morphology of PLA/PBAT/BF Composites

**Figure 3:**
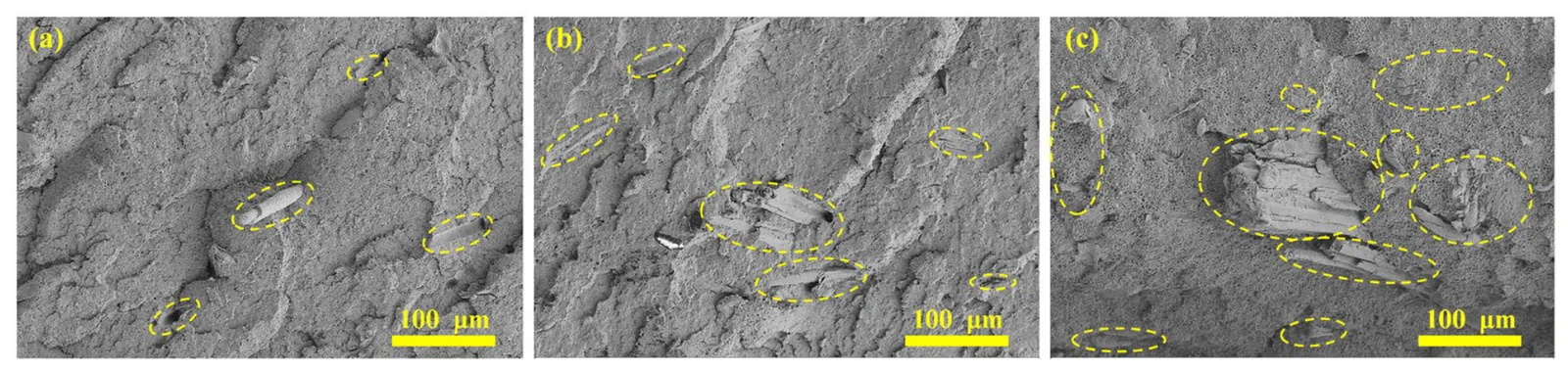
SEM micrographs of cryo-fractured surfaces for (**a**) PLA/PBAT/BF-2, (**b**) PLA/PBAT/BF-6, and (**c**) PLA/PBAT/BF-10 composites.

Figure 3 shows cryo-fractured SEM micrographs of PLA/PBAT/BF composites. For PLA/PBAT/BF-2, BFs disperse relatively evenly in the PLA/PBAT matrix (Figure 3a). When BF loading increases to 6 and 10 wt.%, obvious fiber agglomeration can be clearly observed Figure 3b,c. Individual well-dispersed BF and local BF aggregates coexist in these two samples, yet a continuous percolated fiber network is not formed in the matrix. In addition, PBAT exists as fine spherical droplets embedded in the PLA continuous phase, and the fracture morphology indicates favorable interfacial interaction between PLA and PBAT phases. This observation may be interpreted as an indication that zinc acetate effectively catalyzed the transesterification reaction between PLA and PBAT, thereby leading to improved compatibility between the two polymers.

The morphological observations from SEM match well with the rheological results from Figure 2. The local BF aggregation seen in SEM accounts for the slight deviation of Han curves at high fiber content. Meanwhile, the absence of a fully connected fiber network explains the moderate increase in complex viscosity and storage modulus in melt rheology. The well-maintained PLA/PBAT interfacial structure in SEM also supports the conclusion that the polymer matrix miscibility remains intact, as suggested by the Han plot analysis.

### 3.3. Mechanical Properties of PLA/PBAT/BF Composites

**Figure 4:**
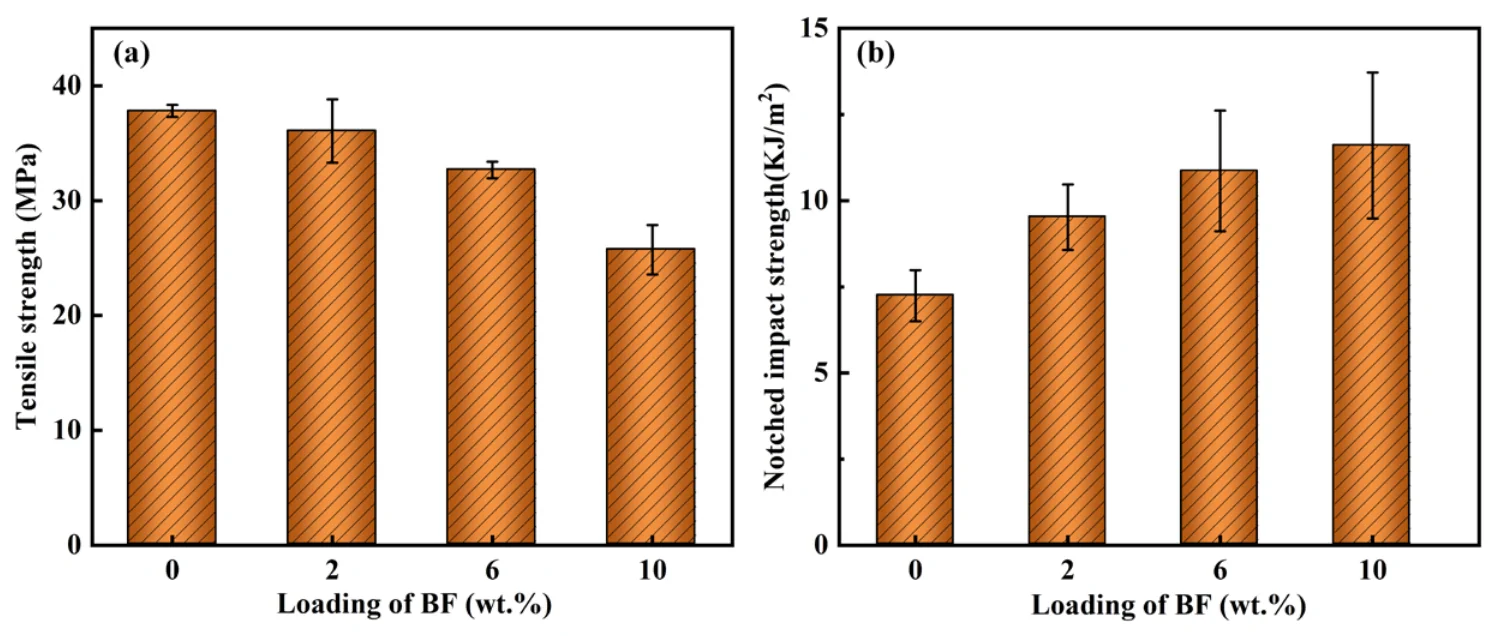
(**a**) Tensile strength and (**b**) notched impact strength of PLA/PBAT and PLA/PBAT/BF composites.

Figure 4a shows the tensile strength of PLA/PBAT/BF composites with different BF loadings (0, 2, 6, 10 wt.%) at a fixed PLA/PBAT weight ratio of 80/20. As BF loading increases, tensile strength gradually declines from 37.0 MPa to 36.0, 33.0, and 26.0 MPa for 2, 6, and 10 wt.% BF, respectively. This reduction mainly arises from progressive BF agglomeration at higher filler content. Combined with SEM observations, weak interfacial adhesion between BF and the PLA/PBAT matrix fails to efficiently transfer tensile stress. Thus, BF cannot exert an expected reinforcing effect under tensile loading, while PBAT serves as a softener for the PLA matrix.

Figure 4b presents the notched impact strength. In contrast to tensile behavior, the impact strength increased with BF addition. The value rose from 7.2 KJ/m^2^ for the neat blend to 11.6 KJ/m^2^ at 10 wt.% BF. This enhancement can be attributed to two synergistic factors. First, the ductile PBAT phase toughens the brittle PLA matrix, enabling plastic deformation during impact [24,25]. Second, the incorporated bamboo fibers effectively hinder crack propagation and absorb impact energy through fiber pull-out and fracture mechanisms [26,27]. Consequently, despite the decline in tensile strength, the incorporation of BF significantly improves the impact toughness of the PLA/PBAT/BF composites. These results demonstrate that the combined use of a transesterification catalyst and bamboo fibers offers a viable strategy to balance stiffness, toughness, and sustainability in bio-based composites.

**Figure 5:**
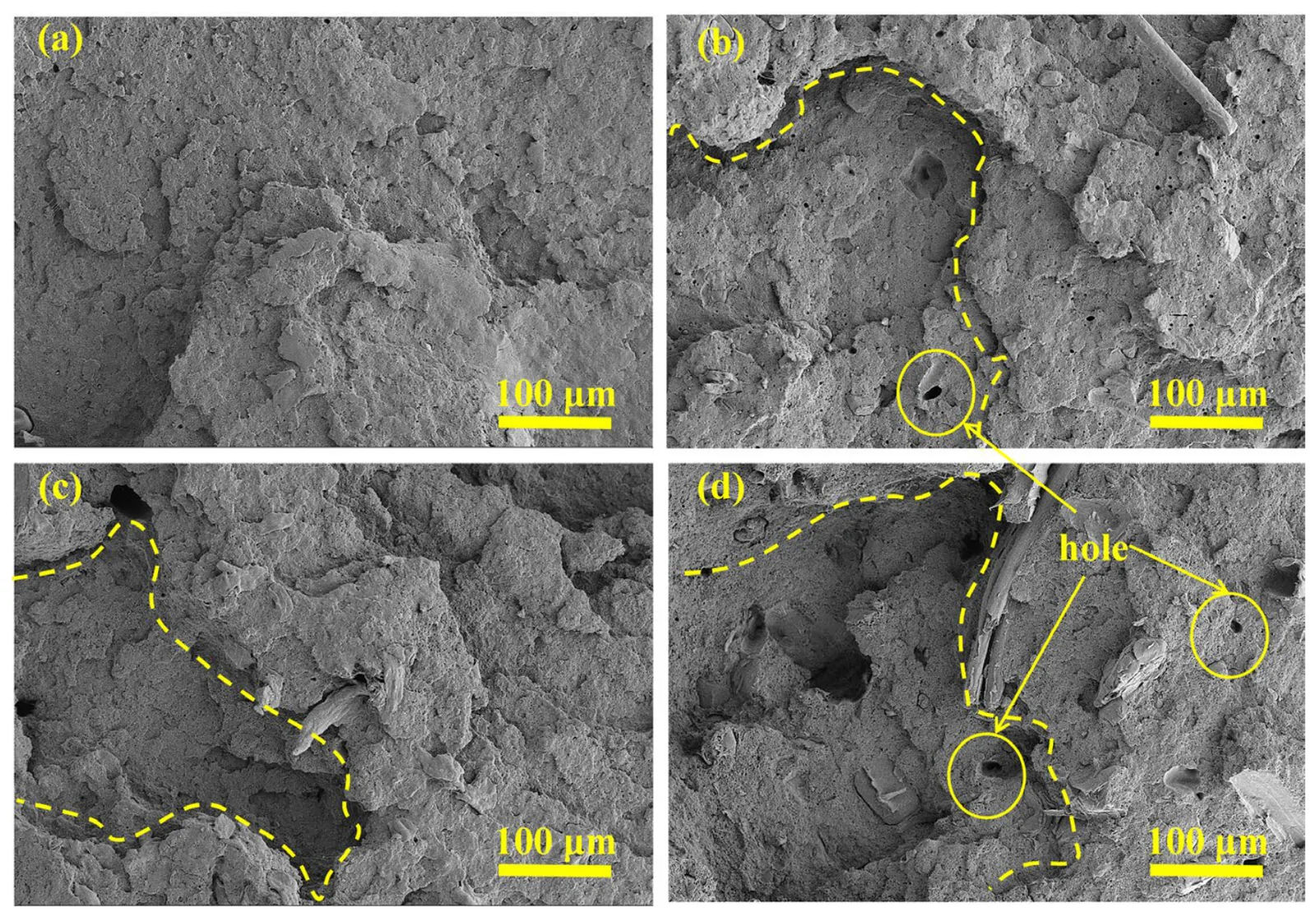
SEM micrographs of cryo-fractured surfaces for (**a**) PLA/PBAT blend, (**b**) PLA/PBAT/BF-2, (**c**) PLA/PBAT/BF-6, and (**d**) PLA/PBAT/BF-10 composites.

To elucidate the underlying toughening mechanism, the impact fracture surfaces were examined by SEM. Figure 5a shows the fracture surface of the neat PLA/PBAT blend, which appears relatively smooth, characteristic of brittle failure [28,29]. In contrast, the composites with BF (Figure 5b–d) exhibit progressively rougher surfaces with ”dimples”. For PLA/PBAT/BF-2 (Figure 5b), small and shallow dimples begin to appear, suggesting local plastic deformation. As BF loading increases to 6 wt.% (Figure 5c) and further to 10 wt.% (Figure 5d), the dimples become more abundant, deeper, and more uniformly distributed across the matrix.

These dimples are typical of ductile fracture and result from the pull-out and debonding of BFs during impact loading. The fiber pull-out process not only dissipates energy through friction but also generates micro-voids that subsequently coalesce into larger voids, absorbing substantial impact energy [30]. Moreover, the presence of BF effectively hinders crack propagation by forcing cracks to deviate or bifurcate, as evidenced by the tortuous fracture paths. The combination of increased impact strength and the formation of a dimple-dominated morphology confirms that the toughening mechanism is primarily energy dissipation via fiber pull-out, interfacial debonding, and matrix plastic deformation, with PBAT further contributing to matrix ductility.

### 3.4. 3D Printing of KO

**Figure 6:**
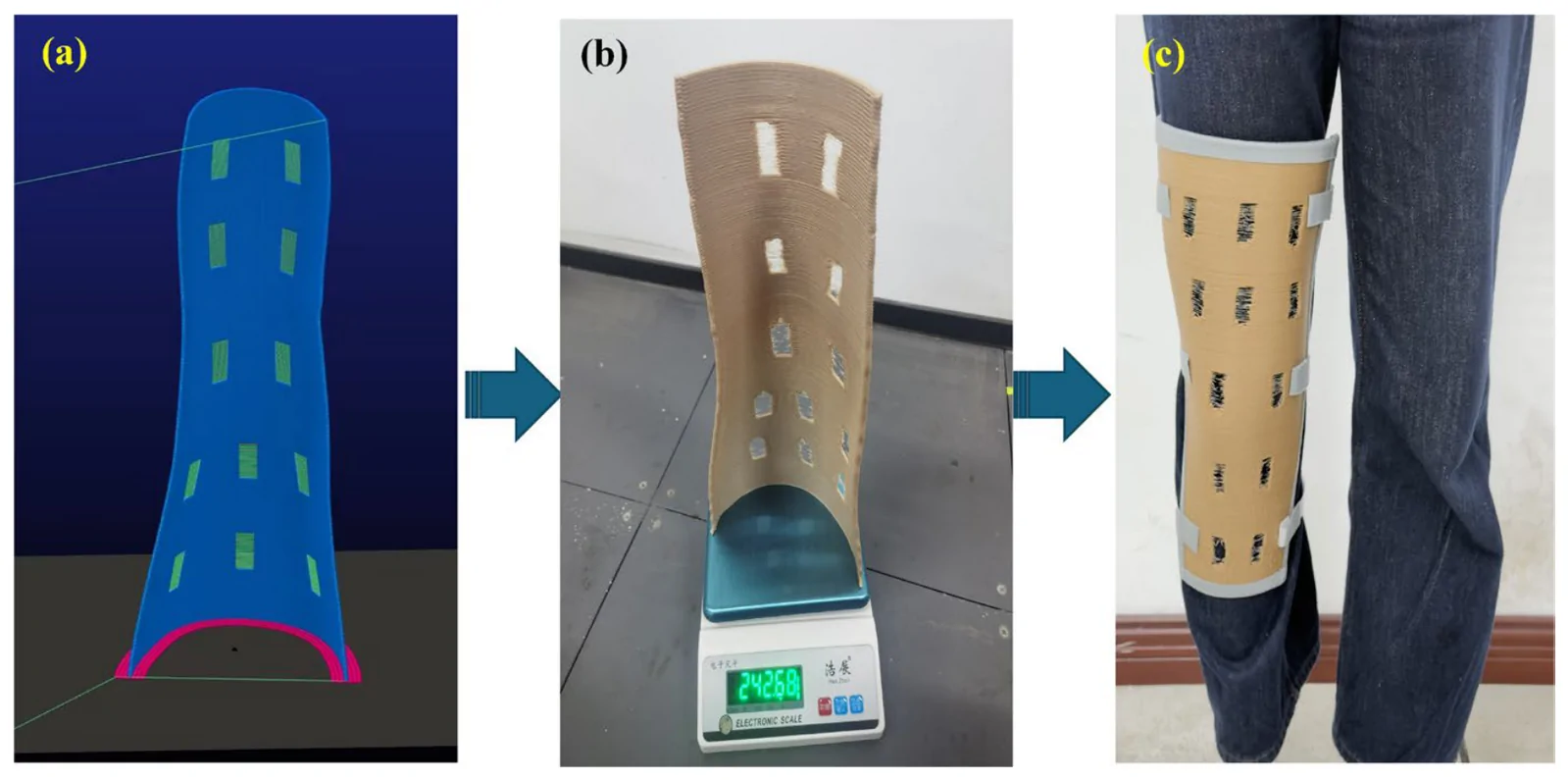
(**a**) Digital model, (**b**) image of 3D-printed PLA/PBAT/BF knee orthosis, and (**c**) image of the knee orthosis worn for demonstration.

Figure 6a presents the digital model of a knee orthosis (KO) created using CAD software as part of a subject-specific design workflow, illustrating the geometric customization capability of the approach. Figure 6b shows the corresponding 3D-printed prototype shell, whose mass was approximately 243 g. This low mass indicates the light-weighting potential of the PLA/PBAT/BF composite combined with a lattice design, which reduces material usage compared with typical reported weights of conventional metal- or plastic-based orthoses, often exceeding 500 g [31,32]. However, these results should be interpreted as a demonstration of material processability and printability of a prototype shell, not as evidence of structural integrity or functional performance.

Figure 6c shows the first author wearing the prototype for demonstration, providing a visual indication of the shell’s overall geometry and dimensions relative to the lower limb. The subject-specific CAD workflow, based on 3D scanning and modeling, allowed the prototype to be designed according to the wearer’s knee and calf geometry. The shell could also be printed rapidly with relatively little material waste, suggesting a potentially sustainable and user-centered fabrication route for future orthoses [33,34]. However, this photograph does not demonstrate patient comfort, mechanical performance, gait, treatment compliance, or clinical outcomes. Future work should therefore include evaluations of stiffness, cyclic loading, fatigue, deformation, interface pressure, gait, and clinical outcomes to further validate the functional and clinical performance of the orthosis.

### 3.5. Effects of Nozzle Temperature on KO Printability

**Figure 7:**
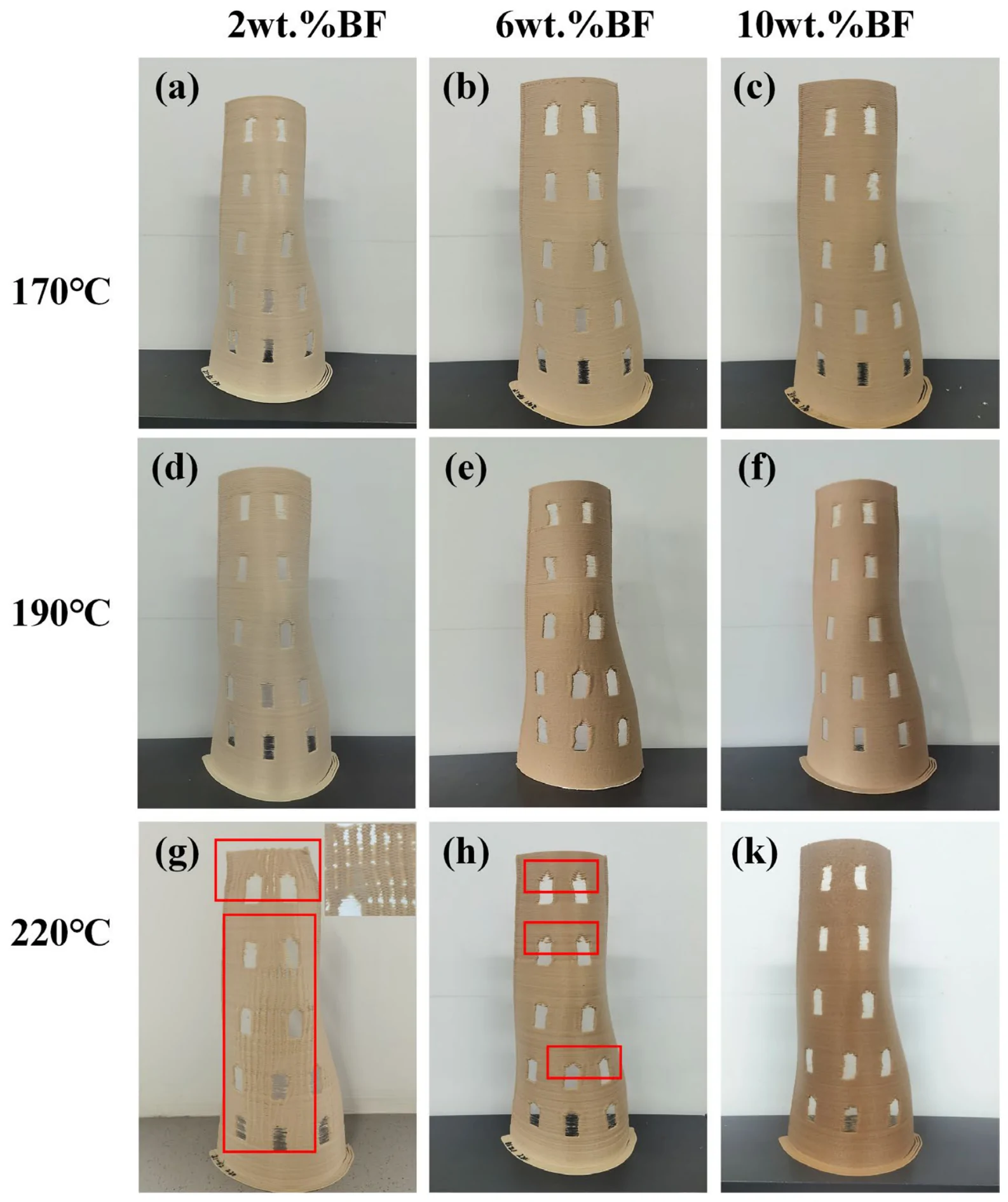
PLA/PBAT/BF-2, PLA/PBAT/BF-6, and PLA/PBAT/BF-10 composite knee orthoses 3D printed at different nozzle temperatures. Printing speed: 30 mm/s (**a**–**h**,**k**).

Figure 7 presents photographs of KO products fabricated from PLA/PBAT/BF composites with three BF loadings (2, 6, and 10 wt.%) using three nozzle temperatures (170, 190, and 220 °C). The printing speed is fixed at 30 mm/s. At 170 and 190 °C, all three composites produced well-defined orthoses without visible defects, indicating sufficient melt flowability and interlayer adhesion within this temperature window. However, at 220 °C, a clear temperature-dependent deterioration in print quality was observed. The PLA/PBAT/BF-2 sample exhibited pronounced surface defects, while the PLA/PBAT/BF-6 sample showed defects specifically at the top edges of the ventilation holes. In contrast, the PLA/PBAT/BF-10 sample maintained good printability even at 220 °C, with no apparent imperfections.

**Figure 8:**
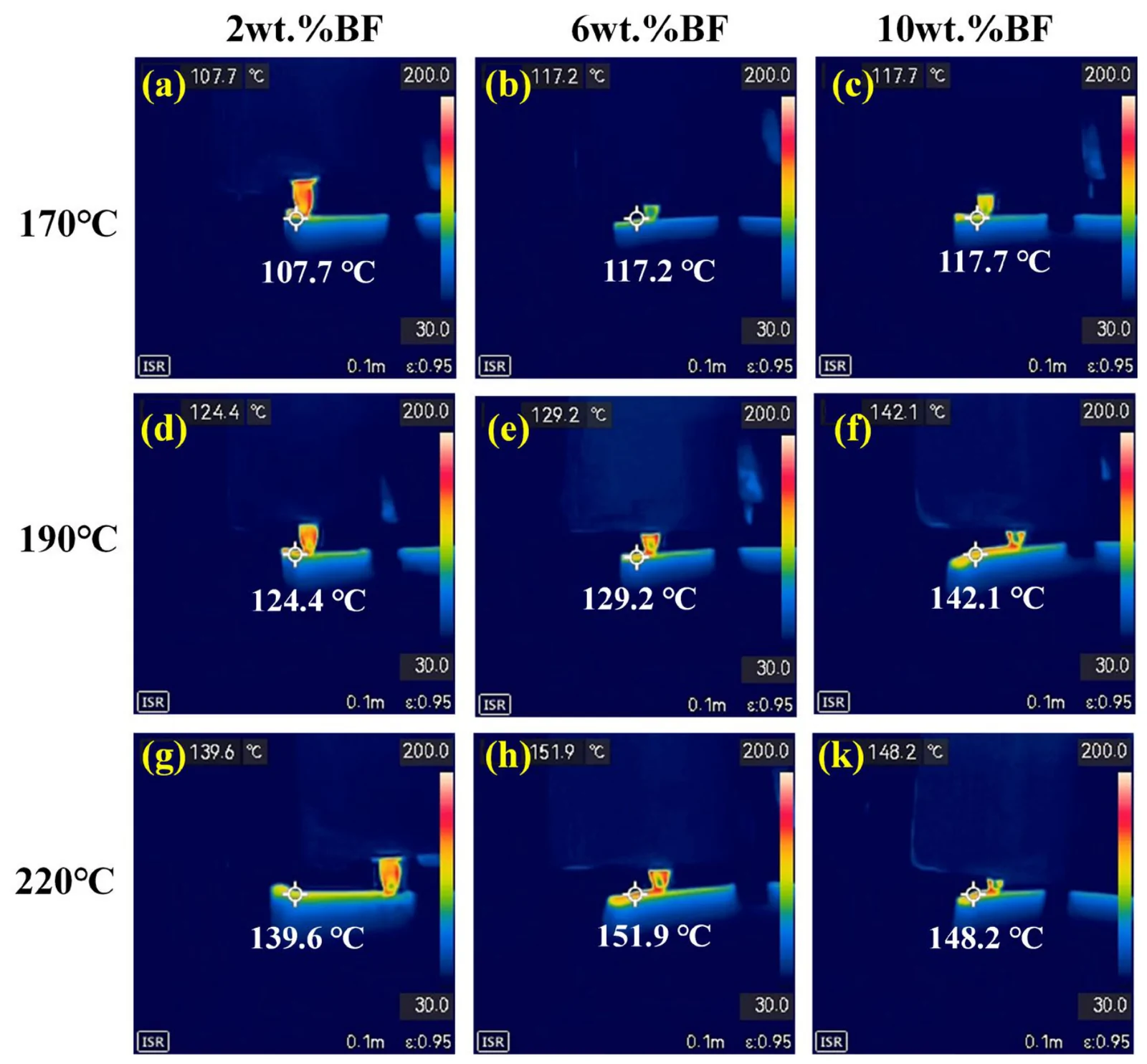
Infrared thermal images of PLA/PBAT/BF composite knee orthoses with different BF loadings during 3D printing (**a**–**h**,**k**).

Figure 8 presents infrared thermographs recorded during 3D printing of PLA/PBAT/BF composites at nozzle temperatures of 170, 190, and 220 °C. As the nozzle temperature increased, the measured surface temperature generally rose for all composites. For instance, at 2 wt.% BF, the center temperature increased from 107.7 °C at 170 °C to 124.4 °C at 190 °C and 139.6 °C at 220 °C, with similar trends for the 6 and 10 wt.% BF composites. At a fixed nozzle temperature, higher BF loadings often led to higher recorded surface temperatures, although the 10 wt.% BF composite showed a slightly lower value than the 6 wt.% BF composite at 220 °C (148.2 °C vs. 151.9 °C). These infrared data mainly reflect relative surface-temperature trends, which may reasonably be associated with differences in heat dissipation. While infrared thermography alone cannot directly quantify melt strength or thermal retention, it offers complementary surface-temperature evidence that is consistent with the printability behavior observed in Figure 7.

The printability results in Figure 7 show that at 220 °C, the 2 and 6 wt.% BF composites exhibited surface irregularities and local collapse, whereas the 10 wt.% BF composite remained visually defect-free. This difference could be associated with temperature-dependent melt strength, since higher temperatures may reduce melt viscosity and dimensional stability [35]. Because the rheological measurements were performed at 190 °C, the proposed relationship among BF loading, melt temperature, melt strength, and printability is best understood as a reasonable mechanism supported by the current results, rather than as a fully established conclusion. Temperature-dependent rheology and direct melt-strength measurements at 170, 190, and 220 °C would provide further validation. Overall, the infrared results supplement the surface-temperature analysis and help interpret the printability differences, indicating a mechanism that warrants further investigation.

### 3.6. Effects of Printing Speed on KO Printability

**Figure 9:**
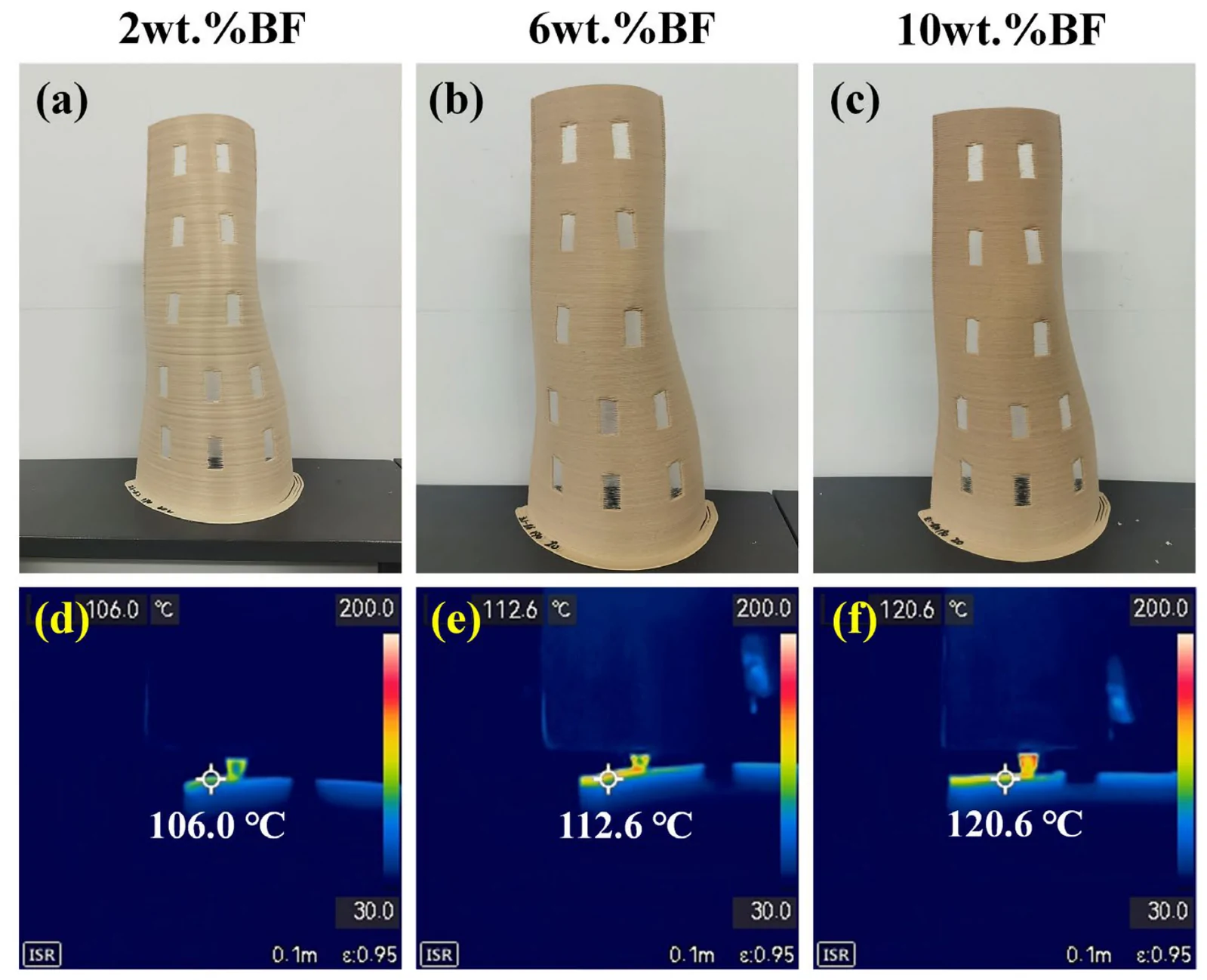
Knee orthoses 3D printed at a speed of 20 mm/s, and infrared thermal images taken during printing (**a**–**f**).

Figure 9a–c show the KO products 3D printed using PLA/PBAT/BF composites with different BF loadings (2, 6, and 10 wt.%), at a nozzle temperature of 190 °C and a relatively slow printing speed of 20 mm/s. Under these conditions, all three composites produced orthoses with high print quality, exhibiting smooth surfaces and well-defined features without apparent defects. The slower printing speed allows each deposited filament more time to cool and dissipate heat to the surrounding environment before the next layer is added [36]. This controlled cooling promotes sufficient solidification of the molten polymer, reduces residual thermal stress, and minimizes warpage or sagging, thus leading to improved geometric accuracy [37].

Figure 9d–f provide the measured temperatures for each composite: 106.0 °C, 112.6 °C, and 120.6 °C for PLA/PBAT/BF-2, PLA/PBAT/BF-6, and PLA/PBAT/BF-10, respectively. The measured temperature increases with higher BF loading, suggesting that BF may influence the thermal behavior of the melt, even at a slow printing speed. At a slower printing speed (e.g., 20 mm/s), the deposited melt has more time to dissipate heat and solidify, which helps maintain printing fidelity across all BF loadings. This effect appears particularly relevant for low-BF formulations, which may be more susceptible to excessive cooling, whereas high-BF compositions exhibit adequate process stability.

**Figure 10:**
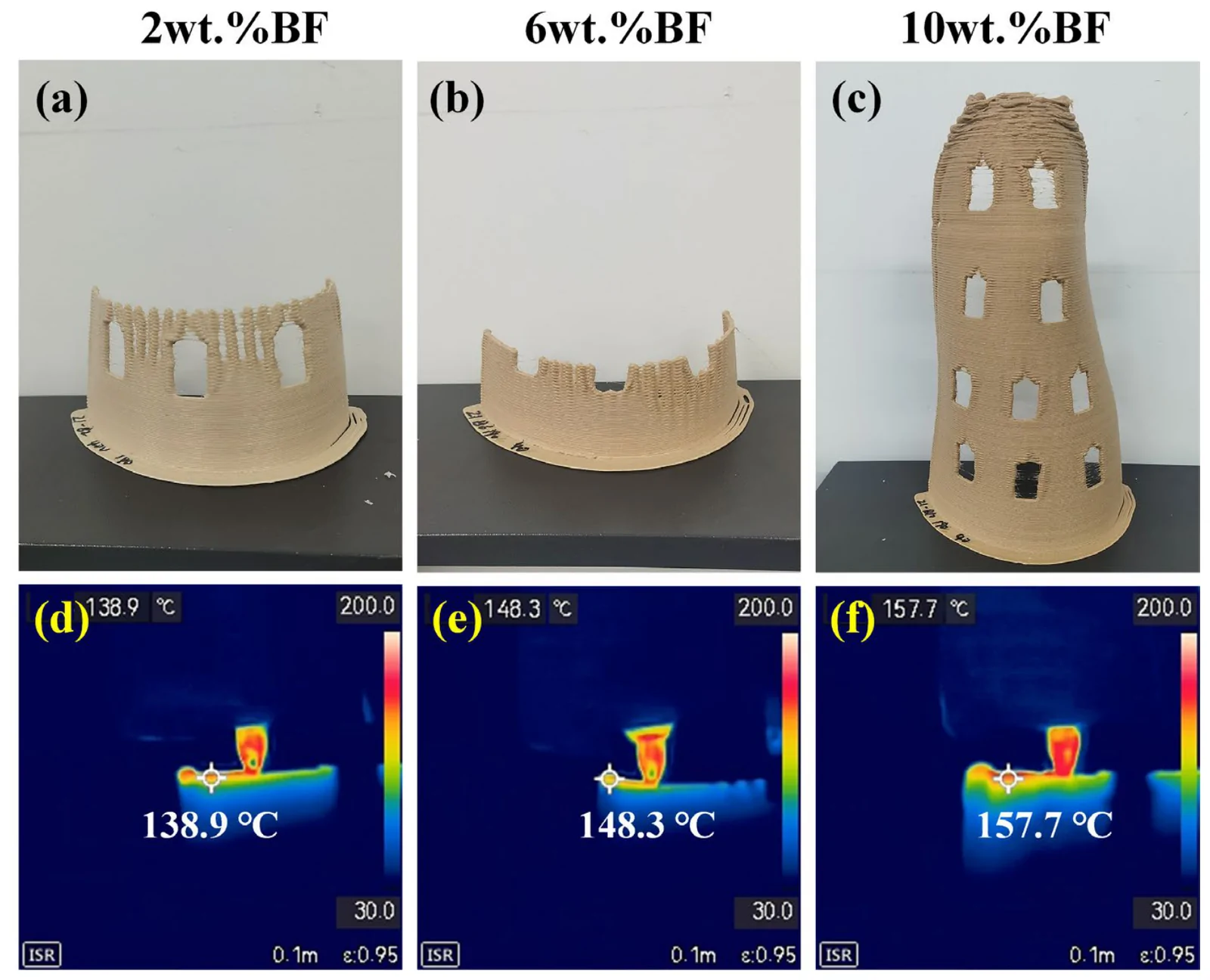
Knee orthoses 3D printed at a speed of 40 mm/s, and infrared thermal images taken during printing (**a**–**f**).

Figure 10 presents the KO products fabricated from PLA/PBAT/BF composites with 2, 6, and 10 wt.% BF using a nozzle temperature of 190 °C, but at a higher printing speed of 40 mm/s. Under these conditions, the printability deteriorated significantly compared to the slower speed (20 mm/s) shown in Figure 9. The PLA/PBAT/BF-2 composite failed completely, and the PLA/PBAT/BF-6 composite also did not print successfully. Even the PLA/PBAT/BF-10 composite, which exhibited good printability at lower speeds, produced an orthosis with noticeable collapse at the top section.

The measured melt temperatures for PLA/PBAT/BF-2, PLA/PBAT/BF-6, and PLA/PBAT/BF-10 under these conditions were 138.9 °C, 148.3 °C, and 157.7 °C (Figure 10d–f), respectively. These values are substantially higher than those recorded at 20 mm/s (see Figure 9d–f), confirming a significant rise in melt temperature with increased printing speed. This is due to the high printing speed (40 mm/s) reduces the residence time of each deposited filament on the build platform, preventing adequate heat dissipation before the next layer is applied. Consequently, the molten material loses dimensional stability, causing sagging, warping, and ultimately print failure. For low BF loadings (2 and 6 wt.%), the melt strength is insufficient to resist deformation under heat accumulation, resulting in complete failure. For the PLA/PBAT/BF-10, the higher fiber loading increases melt strength and provides some structural reinforcement, yet the accumulated heat still causes partial collapse at the top, where the cooling time is shorter.

## 4. Conclusions

This study developed biodegradable PLA/PBAT/BF composites and fabricated lightweight custom-fit knee orthoses via FGF 3D printing. Adding 2, 6, and 10 wt.% BF modified the melt rheology: complex viscosity and storage modulus increased with fiber loading, consistent with restricted chain mobility and enhanced melt elasticity, while Han plots and SEM indicated local fiber aggregation at higher loadings without obvious deterioration in PLA/PBAT compatibility. Mechanically, the BF imparted a stiffness–toughness trade-off: tensile strength decreased from 37.0 to 26.0 MPa, while notched impact strength increased from 7.2 to 11.6 kJ/m^2^, possibly due to fiber pull-out and debonding that impeded crack growth. A subject-specific orthosis weighing 243 g was printed from a 3D body scan, indicating the potential of FGF for lightweight design. Printability depended on printing temperature and fiber loading. Infrared thermography showed that BF elevated the melt-surface temperature; at 220 °C, low-fiber composites exhibited sagging and collapse, whereas the 10 wt.% formulation remained stable. At 40 mm/s, all composites showed printing failure. Under the conditions tested, the optimal processing window was 10 wt.% bamboo fiber at 190–220 °C and 30 mm/s, yielding tough, lightweight, biodegradable orthoses. These results suggest that granule-based FGF is a promising route for personalized orthotic devices, although further studies are needed to validate mechanical performance, long-term durability, and clinical suitability.

## Data Availability

The original contributions presented in this study are included in the article. Further inquiries can be directed to the corresponding authors.
